# Modulating Activity of Vancomycin and Daptomycin on the Expression of Autolysis Cell-Wall Turnover and Membrane Charge Genes in hVISA and VISA Strains

**DOI:** 10.1371/journal.pone.0029573

**Published:** 2012-01-09

**Authors:** Viviana Cafiso, Taschia Bertuccio, Daniela Spina, Simona Purrello, Floriana Campanile, Cinzia Di Pietro, Michele Purrello, Stefania Stefani

**Affiliations:** 1 Unit of Microbiology, Department of Bio-Medical Sciences University of Catania, Catania, Italy; 2 Unit of Genome and Molecular Complex Systems BioMedicine G Sichel, Department Gian Filippo Ingrassia, Catania, Italy; Baylor College of Medicine, United States of America

## Abstract

Glycopeptides are still the gold standard to treat MRSA (Methicillin Resistant *Staphylococcus aureus*) infections, but their widespread use has led to vancomycin-reduced susceptibility [heterogeneous Vancomycin-Intermediate-*Staphylococcus aureus* (hVISA) and Vancomycin-Intermediate-*Staphylococcus aureus* (VISA)], in which different genetic loci (regulatory, autolytic, cell-wall turnover and cell-envelope positive charge genes) are involved. In addition, reduced susceptibility to vancomycin can influence the development of resistance to daptomycin. Although the phenotypic and molecular changes of hVISA/VISA have been the focus of different papers, the molecular mechanisms responsible for these different phenotypes and for the vancomycin and daptomycin cross-resistance are not clearly understood. The aim of our study was to investigate, by real time RT-PCR, the relative quantitative expression of genes involved in autolysis (*atl*-*lyt*M), cell-wall turnover (*sce*D), membrane charges (*mpr*F-*dlt*A) and regulatory mechanisms (agr-locus-*gra*RS-*wal*KR), in hVISA and VISA cultured with or without vancomycin and daptomycin, in order to better understand the molecular basis of vancomycin-reduced susceptibility and the modulating activity of vancomycin and daptomycin on the expression of genes implicated in their reduced susceptibility mechanisms. Our results show that hVISA and VISA present common features that distinguish them from Vancomycin-Susceptible *Staphylococcus aureus* (VSSA), responsible for the intermediate glycopeptide resistance i.e. an increased cell-wall turnover, an increased positive cell-wall charge responsible for a repulsion mechanism towards vancomycin and daptomycin, and reduced agr-functionality. Indeed, VISA emerges from hVISA when VISA acquires a reduced autolysis caused by a down-regulation of autolysin genes, *atl*/*lyt*M, and a reduction of the net negative cell-envelope charge via *dlt*A over-expression. Vancomycin and daptomycin, acting in a similar manner in hVISA and VISA, can influence their cross-resistance mechanisms promoting VISA behavior in hVISA and enhancing the cell-wall pathways responsible for the intermediate vancomycin resistance in VISA. Daptomycin can also induce a charge repulsion mechanism both in hVISA and VISA increasing the activity of the *mp*rF.

## Introduction

Emerging resistance to glycopeptides in Methicillin-Resistant *Staphylococcus aureus* (MRSA) poses a great threat to antimicrobial chemotherapy worldwide. Together with the recent discovery, in 2002, of the first clinical isolate of fully Vancomycin-Resistant *S.aureus* (VRSA) with VAN MIC >128 mg/L [1], numerous other isolates of homogeneous Vancomycin-Intermediate *Staphylococcus aureus* (VISA) or heterogeneous Vancomycin-Intermediate *Staphylococcus aureus* (hVISA) have been isolated worldwide. In these strains, this reduced susceptibility has been attributed to various cell-wall abnormalities, evolving in a multi-step fashion. These abnormalities include accumulation of D-Ala-D-Ala targets due to decreased cross-linking of peptidoglycan [2], the increased proportion of non-amidated muropeptides [3], and decreased alanylation of teichoic acids [4].

VISA does not emerge from vancomycin-susceptible MRSA but from hVISA, as was previously demonstrated: hVISA spontaneously produces VISA cells within its cell population at a frequency of 10^−6^ or above [5] that is the same frequency of hVISA onset from a susceptible background [6].

Recent publications have added to the knowledge of the complex changes taking place in Staphylococci evolving towards the reduced glycopeptide susceptibility phenomenon. A reduced content of Lysyl-phosphatidylglycerol (LPG), synthesized by *fmt*C (also named *mpr*F) encoded protein, has recently been demonstrated affecting some cationic antimicrobial agents including vancomycin (VAN), but also the Ca^2+^- daptomycin (DAP) [7]. In addition, a possible role of the autolytic enzymes has also been suggested, among which the major autolysin *atl* [8], the peptidoglycan hydrolase *lyt*M [9], and the transglycolase *sce*D [10] variously involved in the physiology of the cell-wall as mediators of cell division, autolysis and peptidoglycan turnover.

Moreover, different regulatory loci have been found to be involved in vancomycin intermediate resistance such as agr-locus (accessory gene regulator locus) encoding δ-hemolysin and also considered as its functionality indicator; GraRS and WalKR, both encoding Two-Component Regulatory Systems “TCRSs”, involved in the regulation of *mpr*F/*dlt*A and *atl*/*lyt*M/*sce*D, respectively [11]–[20].

GraRS has been involved in the VISA phenotype, because these lead to increased autolytic rates and a more negative net surface charge, which increase the sensitivity to cationic antimicrobial peptides [15].

Recently, Dubrac *et al.* emphasized the importance of the *wal*KR system in cell-wall metabolism in *S.aureus* [19]. Moreover, Jansen *et al.* reported that *wal*KR was highly up-regulated due to an insertion mutation in the *wal*KR promoter in a VISA clinical isolate, suggesting that the increment of vancomycin resistance was mediated by activation of this system [17]. On the contrary, in a recent paper, Hiramatsu *et al.* reported a deletion mutation in *wal*KR [deletion of 3 nucleotides (CAA) from the position 1111 to 1113] and a truncating mutation in a proteoliytic regulatory gene, *clp*C, responsible for the raised vancomycin resistance in a laboratory derivate strain, but they did not find any significant changes in the expression of *wal*KR in any of the resistant mutants studied. Thus, the cause of raised resistance due to the *wal*KR mutation still remains unknown [21]. Moreover, Delaune *et al.* recently reported on the effect of *wal*KR on cell morphology, showing that *wal*KR depletion could raise the cell-wall thickness of *S.aureus* [20], but this regulatory pathway toward cell-wall thickening remains to be studied.

It has been postulated that reduced susceptibility to VAN can influence the susceptibility to DAP by the thickness of the VISA cell-wall [22]. Furthermore, some of the above mentioned loci, such as *mpr*F and *dlt*-operon, have also been involved in DAP resistance mechanisms of *S.aureus* [23]–[25].

With all this in mind, and using a real time RT-PCR approach, we investigated the relative quantitative expression of some genes involved in autolysis, cell-wall turnover, membrane charges and global regulation, on a sample of hVISA and VISA isolates, in order to define the possible modulating activity of VAN and DAP on the expression of the above mentioned genes involved both in the mechanisms of glycopeptide reduced susceptibility and daptomycin resistance.

## Results

### Phenotype and molecular characteristics of the strains

The strains showed a range of resistance to glycopeptides from fully susceptible (NRS149) to hetero-resistant (Mu3 and CZ1, SS33, 004/210 with PAP/AUC ratio of 1.06, 1.07 and 1.22 respectively) to homogenous intermediate Mu50, both with Macro E-test (MET) and PAP/AUC analyses (gold standard method). All isolates were susceptible to daptomycin with the only exception of Mu50 that shows a daptomycin MIC value of 2 mg/L considered as borderline phenotype. All strains homogeneously belonged to agr-group II and to Clonal Complex 5 (CC5); in particular, three of them were Sequence Type 5 (ST) and the three clinical isolates CZ1, SS33 and 004/210 belonged to the well-known nosocomial ST228. MRSA strains possessed the Staphylococcal Cassette Chromosome *mec* I (SCC*mec* I) and II type (Table 1).

**Table 1:** Strains included in the study.

Strain	Origin	Source	OxacillinResistance	Glycopeptide phenotype	MIC(mg/L)			Macro ETest(mg/L)		MLST/ssc*mec*characterization	agr-group
					V	T	D	V	T		
**NRS149**	NARSA	Nares of nurse	MSSA	VSSA	1	0.5	0.5	1	0.5	ST5	II
**Mu3**	NARSA	Purulent sputum	MRSA	hVISA	0.5	8	1	6	24	ST-5scc-*mec*II	II
**Mu50**	NARSA	Wound Skin/Soft tissue	MRSA	VISA	8	16	2	12	12	ST-5scc-*mec*II	II
**CZ1**	Clinical Isolate	Air pipe	MRSA	hVISA	1	0.5	0.5	4	16	ST-228scc-*mec*I	II
**SS33**	Clinical Isolate	Blood	MRSA	hVISA	0.5	0.5	0.5	4	16	ST-228scc-*mec*I	II
**004/210**	Clinical Isolate	Abscess/pus	MRSA	hVISA	1	0.5	0.5	6	12	ST-228scc-*mec*I	II

Legend: V = Vancomycin, T = Teicoplanin, D = Daptomycin, S = Susceptible, R = Resistant.

### Total autolysis ratio and δ-hemolysin production

Table 2 shows the autolysis ratio, read as an optical density (O.D.) value obtained in drug-free medium cultured strains, demonstrating the greatest total Triton X-100 autolysis (75–76%) in hVISA with respect to the susceptible strain. A lower Triton X-100 autolysis was found in Mu50 and in the clinical strain 004/210 (49 and 60%, respectively).

**Table 2:** δ-hemolysis, Autolysis Ratio, Percentage of Triton X-100 autolysis *vs.* VSSA.

Strain	δ-hemolysinproduction*	Autolysis Ratio(means ± SD)**	Percentage of Triton X-100 autolysis *vs.*VSSA
**NRS149**	++	2.68±0.05	−
**Mu3**	+/−	2.04±0.07	76%
**Mu50**	−	1.33±0.06	49%
**CZ1**	+/−	2.00±0.04	76%
**SS33**	+/−	2.02±0.07	75%
**004/210**	−	1.62±0.07	60%

Legend:

*(++) large δ-hemolysis zone; (−/+) low δ-hemolysis zone; (−) no δ-hemolysis zone.

**measured as the T_0_/T_5_ O.D. ratio ± SD (Standard Deviation).

**Figure 1:**
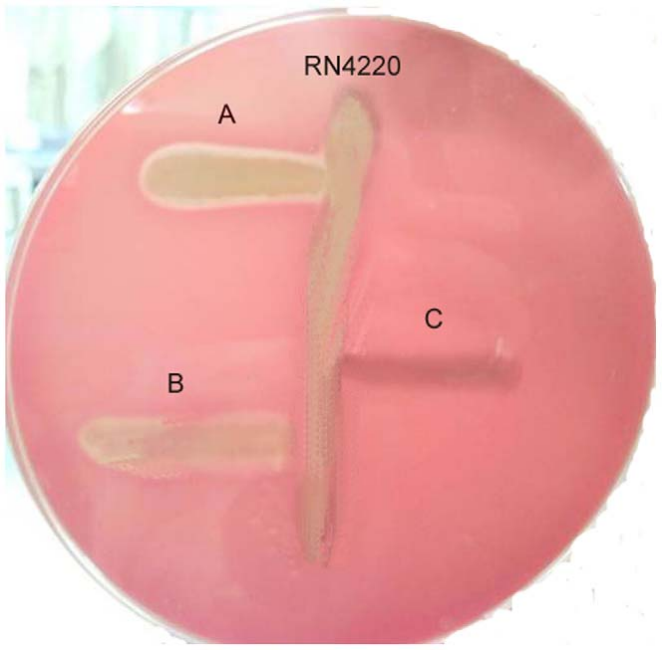
δ-hemolysis assay of prototype microrganisms. A) NRS149 (VSSA); B) Mu3 (hVISA); C) Mu50 (VISA).

Our results evidence a gradient of reduction of the δ-hemolysin production from NRS149 to Mu50. In particular, Mu50 and 004/210 lacked the δ-hemolysis activity whereas NRS149 presented a large zone of hemolysis. hVISA (Mu3, CZ1, and SS33) exhibited, instead, a low δ-hemolysis activity (Figure 1).

### *gra*R and *wal*K mutations

Sequence analysis of *gra*R excluded the presence of the mutation, changing the 1197th Asn in Ser, related to vancomycin resistance in CZ1, SS33, and 004/210. *wal*K sequencing revealed the absence of the truncating mutation of 3 nucleotides (CAA) from the position 1111 to 1113 in all tested strains.

### Relative quantitative expression of some autolytic, cell-wall charge and virulence regulator genes in drug-free conditions

**Figure 2:**
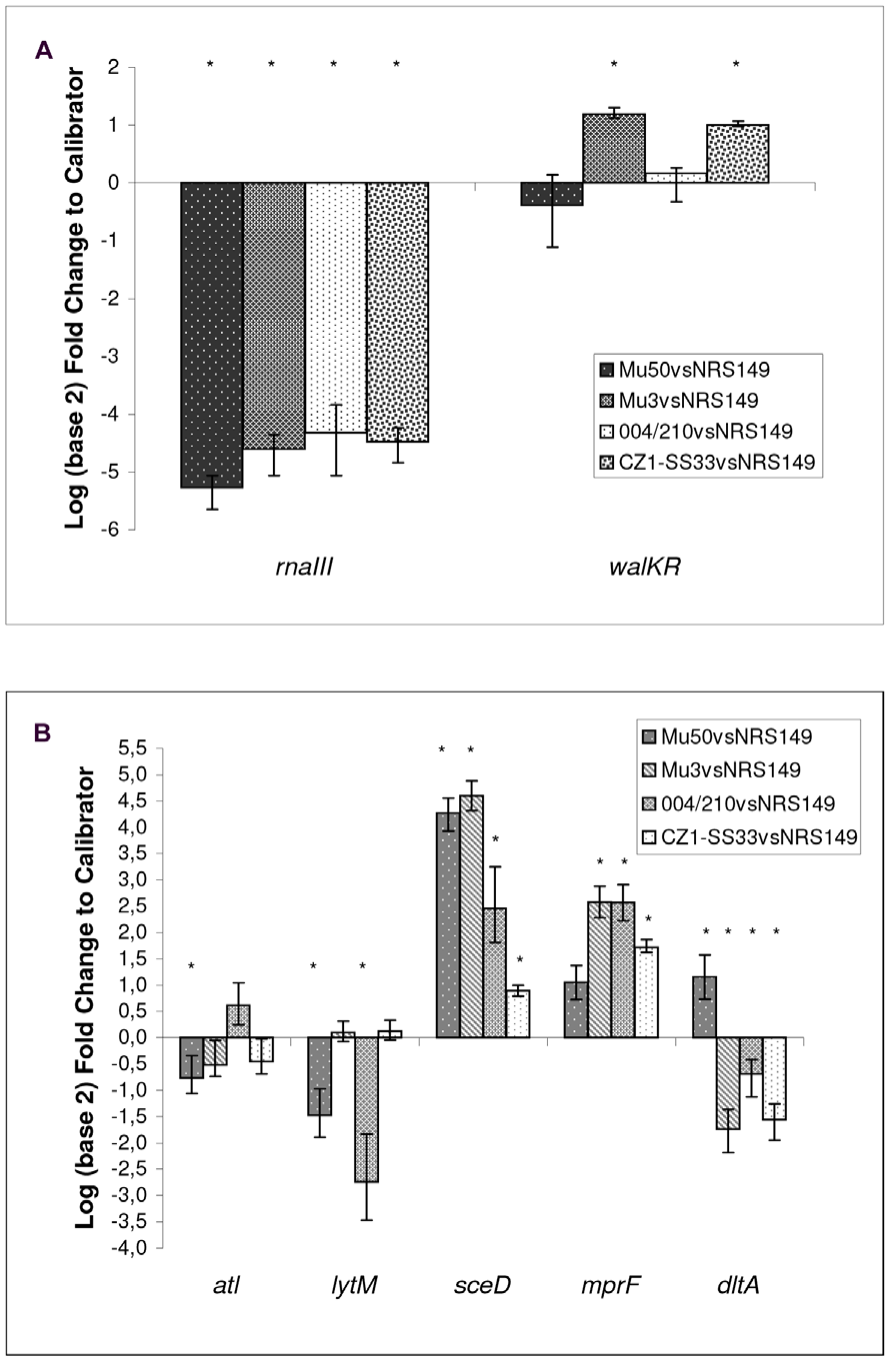
Transcript analysis in drug-free conditions. (A) Relative quantitative expression of the *rna*III and *wal*K regulator genes and (B) of some autolytic (*atl*, *lyt*M), cell-wall turnover (*sce*D) and cell envelope charge genes (*mpr*F, *dlt*A) in hVISA and VISA. Statistically significant difference between sample *vs.* VSSA, p<0.05, are indicated with *.

Figure 2A, 2B and Table S1 show the relative quantitative expression of autolytic, cell-wall charge and virulence regulator genes considered in the study, as a ratio of transcripts of hVISA and VISA *versus* VSSA, in drug-free conditions.

The virulence regulator, *rna*III, showed significantly decreased fold changes in Mu3, CZ1, SS33 and 004/210, and more so in Mu50 with respect to NRS149. This behavior was perfectly comparable to the phenotypes observed in hemolysis assays (Table 2).

No significant differences were observed, in all tested isolates, in the expression of the regulator *gra*RS *versus* NRS149 (data not shown). Furthermore, *wal*K was significantly up-regulated only in Mu3-CZ1-SS33 *versus* NRS149. On the contrary, no significant *wal*K expression changes were found in Mu50 and 004/210 with respect to NRS149.

Our experiments of *atl* expression highlighted a down-regulation of this gene in Mu50 with respect to NRS149, whereas statistically significant differences were not detected between the remaining strains. The relationship between the *lyt*M transcript amounts was similar to the *atl* one, with the exception of 004/210 showing a down-regulation in the same way as in Mu50. The analysis of *sce*D transcription showed an up-regulation in all studied strains with respect to NRS149, and a higher expression in Mu3 and Mu50.

With regard to the two genes involved in cell-wall charge, *mpr*F expression showed an up-regulation of its transcription in Mu3, CZ1, SS33 and 004/210, whereas Mu50 presented no significant changes with respect to NRS149. *dlt*A expression presented an up-regulation of its transcription only in Mu50 with respect to NRS149.

### Relative quantitative expression of some autolytic, cell-wall charge and virulence regulator genes with VAN and DAP

**Figure 3:**
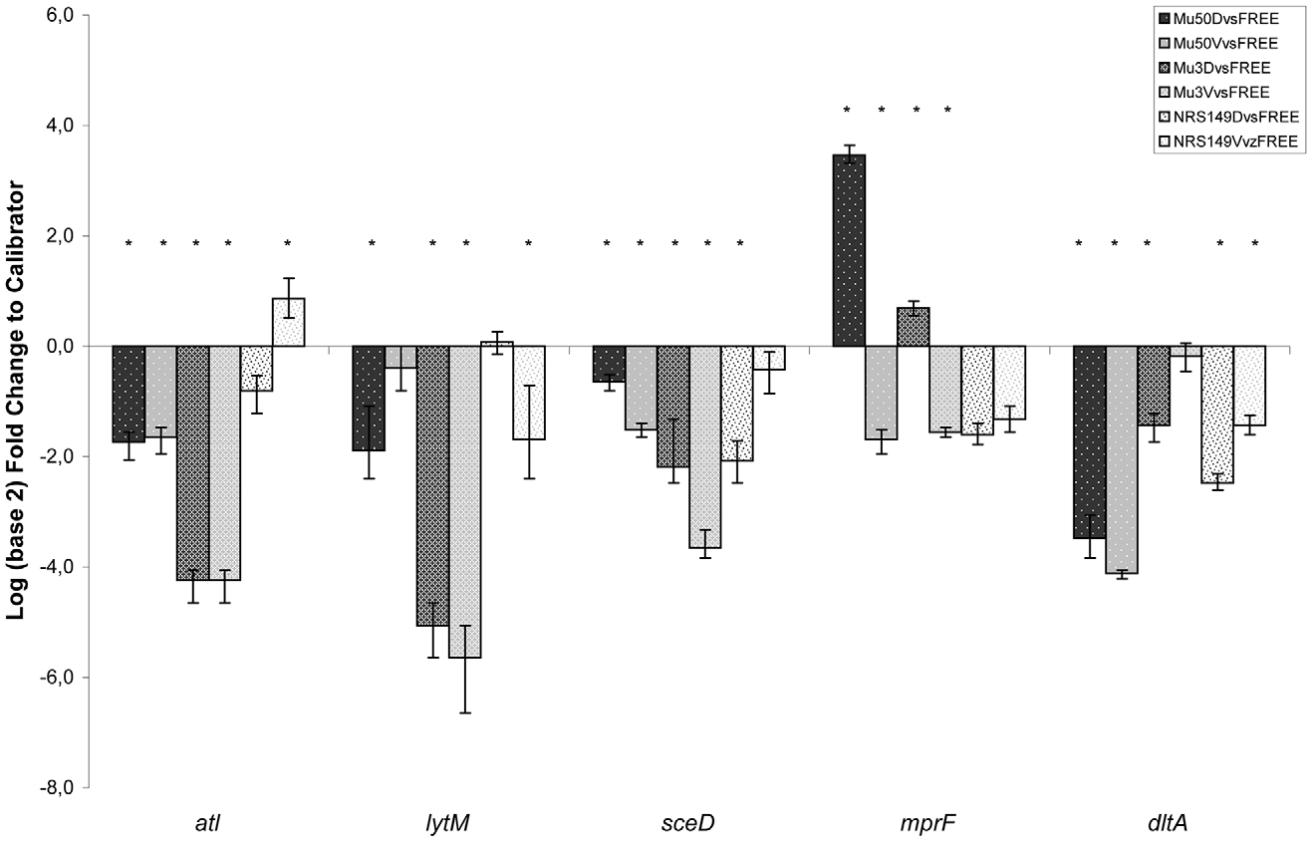
Transcript analysis in presence of vancomycin and daptomycin. Relative quantitative expression of some autolytic (*atl*, *lyt*M), cell-wall turnover (*sce*D) and cell envelope genes (*mpr*F, *dlt*A) of VSSA, hVISA and VISA with VAN (V) and Ca^2+^-DAP (D) *vs* drug-free conditions (FREE). Statistically significant difference between sample *vs.* free drug conditions, p<0.05, are indicated with *.

The picture of relative quantitative expression in the presence of sub-inhibitory concentrations of VAN and DAP showed the influence of these antimicrobials on the expression of autolytic, cell-wall charge, and regulatory genes in NRS149, Mu3 and Mu50 (Figure 3, Table S2).

No significant differences in *rna*III and *gra*RS expression were observed in the presence of VAN and DAP in the growth medium in all of the studied *S.aureus* strains (data not shown). Only *wal*K had a significant down-regulation in Mu50 with both antimicrobials (Table S2).

In the presence of VAN and DAP, Mu50 and Mu3 showed a very low transcription level of the *atl* gene with respect to the drug-free conditions. Moreover, *atl* expression ratio between the two strains was lower in Mu50 than Mu3 both with VAN and DAP.

With both antimicrobials, the amount of *lyt*M transcripts was similar to the *atl* ones both in Mu3 and Mu50, whilst no significant *lyt*M expression changes were observed in Mu50 with VAN *versus* Mu50 in drug-free conditions. Despite the different activity on the *lyt*M expression of the two antimicrobials on Mu3 and Mu50, a similar ratio of *lyt*M transcripts was found between these isolates in the presence of VAN and DAP.

A strong *sce*D down-regulation *versus* drug-free conditions was induced by DAP, and more so by VAN, in Mu3 and Mu50. Furthermore, the *sce*D transcript ratio showed a down-regulation in Mu3 both with VAN and DAP with respect to Mu50.

DAP determined an increase of the *mpr*F transcripts in Mu3 and more so in Mu50. In the presence of VAN, instead, a strong down-regulation of *mpr*F transcription was evident in both strains. With the two antimicrobials, no significant differences in the *mp*rF expression were found comparing the two strains with DAP and VAN.

*dlt*A down-regulation was shown by VAN and DAP in both Mu3 and Mu50 with respect to strains grown in drug-free conditions, with the only exception of Mu3 in the presence of VAN in which no significant changes were found with respect to drug-free conditions. A higher *dlt*A transcription was always found in Mu50 than Mu3 in the presence of DAP. The addition of VAN in the growth medium showed no significant differences in the *dlt*A transcription level between Mu3 and Mu50.

In vancomycin susceptible strain NRS149, the analysis of quantitative transcription profile of autolysin, cell wall turnover and charge genes showed that VAN down-regulated *lyt*M and *dlt*A, whereas only *atl* was up-regulated. No significant changes with respect to drug-free conditions were found in the amount of *sce*D and *mpr*F transcripts. Furthermore, DAP significantly down-regulated *atl*, *sce*D, and *dlt*A, but no significant changes were found for the other tested genes (Figure 3, Table S2).

## Discussion

Glycopeptide reduced susceptibility is a multifactorial event due to several biological factors including thickened cell-wall, reduced cell-wall turnover and autolysis, and increased cell-wall synthesis that lead to reduced vancomycin access to its active site, the division septum [3], [12]. Although the phenotypic changes of hVISA/VISA strains have been the main topic of numerous papers that have attempted to clarify this behavior, molecular mechanisms responsible for the different phenotypes are still not clearly understood.

Two different pathways can be involved in cell-wall thickening i.e.: i) the production of an excess amount of peptidoglycan or; ii) reduced peptidoglycan turnover and cell lysis [22]. For these reasons, we studied the expression levels of different enzymes, selected on the basis of previous proteomic and expression studies, involved in cell-wall expansion, turnover, growth, and cell separation, cell-envelope charge and regulatory mechanisms, in particular: i) agr-locus, WalKR, and GraRS; ii) the *atl* gene, encoding a bi-functional enzyme, with an amidase and a glucosaminidase domain that represents the most predominant peptidoglycan hydrolase of *S.aureus*; iii) the *lyt*M gene, encoding a Gly-Gly endopeptidase; and iv) the *sce*D gene (SAV 2095 *sce*D-like gene) encoding a lytic transglycosylase [11]–[20], [26]–[33].

A loss of agr-functionality in *S.aureus* has also been linked with glycopeptide reduced susceptibility [13]. We observed progressive attenuated hemolytic properties between hVISA and VISA when plated on sheep blood agar plates. In particular, hVISA produced a low amount of delta-hemolysin at 24 h, whereas VISA did not produce it at all, as shown by the absence of hemolysis at the interface with RN4220. Since *hld* (the gene encoding delta-hemolysin) and its promoter are intact in Mu3 and Mu50, the lack of delta-hemolysin expression was most likely due to the loss of *agr* functionality in agreement with previously published data [14], [34]. Our studies on *hld* expression indicate a progressive down-regulation of this gene with respect to VSSA, both in h-VISA and VISA, correlating completely with the hemolytic phenotype observed [12]. It is also important to underline that these strains can be less toxigenic than VSSA, since they did not activate the regulatory mechanisms leading to the production of the main toxin genes, and more in general, of different virulence determinants such as *spa*, encoding protein A [12]. Therefore, our findings provide experimental support for the use of the δ-hemolytic assay as a biomarker to easily and rapidly identify isolates with reduced vancomycin susceptibility as recently presented at ECCMID 2011 Congress, Milan, by our group.

An increased sensitivity to cationic antimicrobial peptides, such as vancomycin and daptomycin, has been attributed to mutations in *gra*RS that lead to increased autolytic rates and a more negative net surface charge [15]. Our studies on *gra*RS, in contrast with results previously published [15], [16], and based on statistically significant data found neither the presence of *gra*RS drug-resistance related mutations nor a significant up-regulation of *gra*RS, involved in the regulation of *mpr*F and *dlt*A expression, in all the studied isolates and in all conditions (drug-free and in the presence of VAN and DAP) (data not shown).

Regarding the presence of resistance-related mutations and the WalKR expression, we found no deletion of 3 nucleotides (CAA) from the position 1111 to 1113 in *wal*K in the studied isolates and, in drug-free conditions, we found an increased *wal*K expression in hVISA, relating to a high Triton X-100 autolysis and an *atl*/*lyt*M expression profile similar to VSSA. On the contrary, a low Triton X-100 autolysis and a decrement of *atl*/*lyt*M transcription, correlating with no increment of *wal*K transcripts, was found in Mu50. Thus, we consider that, in VISA, the reduction of the main autolysin activity can be related to a low *wal*K expression. In addition, DAP and VAN can enhance these molecular traits of the VISA phenotype.

As concerns the autolytic activity, our data show that, in drug-free medium, the VISA strain (Mu50) is constitutively characterized by a low Triton X-100 autolysis ratio, a reduced expression of *atl* and *lyt*M with respect to hVISA and, obviously, VSSA, suggesting that a reduction of *atl* and *lyt*M expression can contribute to the characteristic low autolysis ratio that plays a key role in the VISA mechanism of resistance. On the contrary, in hVISA, our experiments showed a Triton X-100 autolysis ratio and *atl* and *lyt*M expression levels similar to those of VSSA, suggesting that *atl*- and *lyt*M-mediated cell lysis is not a crucial point in the mechanism of heterogeneous vancomycin reduced susceptibility. Our analysis of gene expression, on drug-induced growth, leads us to important new considerations. These antimicrobials exhibit the same modulating activity on the expression of *atl* both in Mu3 and Mu50, down-regulating both strains but with a stronger effect on Mu3, shifting Mu3 towards a behavior that is more similar to Mu50. This consistent reduction of *atl* expression was “drug-induced” by both VAN and DAP in Mu3, and “drug-enhanced” in Mu50. Similar considerations can be made for the *lyt*M down-regulation that would result in a “drug-induced effect” by both antimicrobials in Mu3. Furthermore, we evidenced that VAN and DAP can determine in NRS149 a down-regulation of *lyt*M and *atl* respectively, highlighting that these antimicrobials can also induce in VSSA the molecular traits leading towards a vancomycin-reduced susceptible phenotype. Thus, VAN and DAP induce, also in VSSA, the expression and consequently the synthesis of enzymes involved in the cell-wall architecture, responsible for the mechanisms of glycopeptide reduced susceptibility.

An abundance of *sce*D transcripts is a distinctive feature both in hVISA and VISA, grown in drug-free conditions, correlating with an enhanced cell-wall turnover and the presence of an altered cell-wall structure, in agreement with results obtained by other authors [35].

VAN and DAP also showed a comparable activity on *sce*D expression. VAN and DAP addition strongly down-regulated *sce*D transcription in Mu3 and in Mu50, in contrast with other findings [10]. Thus, *sce*D over-expression is a constitutive feature of the hVISA and VISA phenotype.

Genes involved in the net positive charge of the cell-wall and cell membrane i.e. *mprF* and *dlt*A, were also identified as genetic determinants that mediate both vancomycin reduced susceptibility and daptomycin resistance in *S.aureus*. *mpr*F is involved in: i) Lys-PG synthesis (a major component of the cell-wall), changing the content of the cell-wall by reducing the negative charge of the cell membrane, and regulating the cell-wall cycle [36]; ii) the translocation of positively-charged phospholipids to the outer membrane leaflet.

The *dlt*ABCD operon controls the D-alanylation of wall teichoic acids in response to antimicrobial challenge, increasing the positive charge of the cell-wall that determines a repulsion mechanism towards positive charged molecules [4], [37], [38] but also indirectly regulates the activity of the autolytic system since a decrease of the cell-wall positive charge accelerates autolysin activity [4], [39].

At the basal level, an over-expression of *mpr*F in hVISA and *dlt*A in VISA, responsible for a repulsion resistance mechanism, was found. Thus, the D-alanylation of teichoic acids is induced in VISA, while the Lys-PG synthesis is induced in hVISA. Our results suggest that the charge repulsion mechanism, responsible for the poor binding of cationic antibacterials in hVISA and VISA strains, follows two different pathways i.e. the Lys-PG synthesis in hVISA and the D-ala teichoic acid synthesis in VISA. In addition, the increased expression of *dlt*A in VISA also determines a greater amount of D-ala wall teichoic acids that could be also related with the reduced autolysis ratio that characterizes the VISA phenotype. This is also in agreement with the high autolysis ratio and the absence of *dlt*A up-regulation found in the hVISA phenotype. No VAN and DAP inducing activity was found on *dlt*A expression both in Mu3 and Mu50.

As stated before, DAP but not VAN showed a modulating activity only on *mpr*F transcription determining an up-regulation both in Mu3 and Mu50. Thus, Mu50 and Mu3 *mpr*F over-expression would result in a “drug-enhanced” effect in Mu3 and in a “drug-induced” effect in Mu50 demonstrating that *mpr*F is, in some way, involved in the development of the DAP non-susceptible phenotype.

In independent studies [23], [25] different *mpr*F point mutations, accumulated in a specific region of the N-terminal encoding the hydrophobic part of the junction of the Lys-PG synthase and flippase domains [40], were repeatedly found in clinical DAP non-susceptible isolates but it seems that the presence of such mutations conferring a hypothetical gain in function phenotype, is not sufficient to explain this complex phenomenon.

In fact, evaluating contrasting data reported in two different studies, it emerges that the DAP non-susceptible strains, possessing the S295L substitution, can present either a *mpr*F over-expression or its down-regulation associated with an enhanced expression of the *dlt*-operon. This was also found in our results in which a down-regulation of *mpr*F associated with an enhanced expression of the *dlt*-operon was found in Mu50, which shows a borderline DAP MIC value of 2 mg/L and did not present the *mpr*F mutation that results in a change of S295L. Thus, in the VISA strain the cation charge repulsion was due to the alalynation of teichoic acid instead of the Lysinylation of PG. Moreover, no VAN and DAP inducing activity was found on *dlt*A expression.

**Figure 4:**
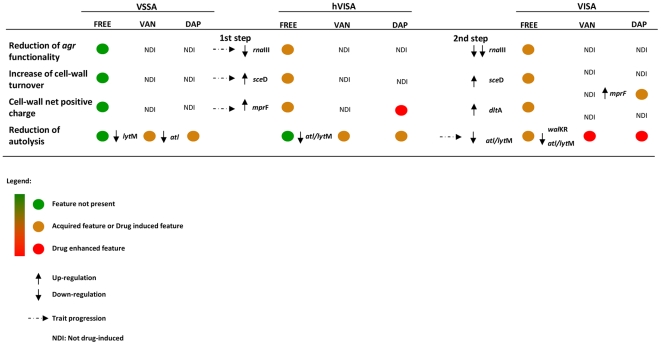
Trait progression involved in the development of the different vancomycin reduced-susceptible phenotypes. Green spot = Feature not present, Orange spot = Acquired feature or Drug induced feature, Red spot = Drug enhanced feature; Up arrow = Up-regulation, Down arrow = Down-regulation, Dotted arrow = Trait progression, NDI = Not drug-induced.

In conclusion, hVISA and VISA exhibit common constitutive features linked with the intermediate level of resistance i.e. an increased cell-wall turnover, an increased positive cell-wall charge responsible for a repulsion mechanism towards VAN and DAP, and a reduced agr-functionality. These properties could represent the key elements that distinguish hVISA and VISA from VSSA. In particular, the VAN reduced susceptibility of hVISA seems to be caused by an increased cell-wall turnover via *sce*D over-expression and a charge repulsion mechanism mediated by *mpr*F up-regulation, while it seems that VISA emerges from hVISA only when a reduced autolysis ratio caused by a down-regulation of major autolysin genes (*atl* and *lyt*M) and a reduction of the negative net cell envelop charge via *dlt*A over-expression is established (Figure 4). VAN, and more so DAP, acting with a similar modulating mechanism both on Mu3 and Mu50, can influence their cross-resistance mechanisms promoting Mu50 behavior in Mu3 and enhancing the cell-wall molecular mechanisms responsible for the vancomycin intermediate resistance in Mu50. DAP, in addition, can induce a charge repulsion mechanism both in Mu3 and Mu50 acting on the *mp*rF gene (Figure 3, Figure 4).

## Materials and Methods

### Strains

The *S.aureus* strains used in this study were NRS149 (VSSA), Mu3 (hVISA), and Mu50 (VISA) kindly supplied by NARSA (Network on Antimicrobial Resistance in *Staphylococcus aureus*) (www.narsa.net), considered as prototypes and controls of VSSA, hVISA and VISA phenotypes both in phenotypic and molecular experiments and three clinical isolates collected in our laboratory CZ1, SS33, and 004/210. The source and relevant characteristics of the bacterial strains studied are listed in Table 1.

### MICs and Macro Etest

MIC determination for glycopeptides and daptomycin was performed according to Clinical and Laboratory Standards Institute (CLSI) guidelines [41].

The Macro Etest (MET) procedure was performed growing all clinical isolates overnight to a 2.0 McFarland standard in Mueller Hinton broth. A 100 µL sample was plated and streaked onto BHI agar and vancomycin and teicoplanin Etest strips were applied (AB BIODISK, Solna, Sweden). Plates were incubated for 48 h at 37°C and were then evaluated for growth following the manufacturer's instructions (EAS003; AB BIODISK) [42]. Mu3 (hVISA), Mu50 (VISA) and ATCC 29213 (VSSA) were used as control strains.

### Population analysis profile/area under the curve analysis (PAP/AUC)

The PAP/AUC procedure was performed as previously published [42]. Briefly, colonies from cultures grown overnight on Tryptic Soy Agar were inoculated into Tryptic Soy broth. Following incubation for 24 h, dilutions of 10^−3^ (10^5^ CFU/mL) and 10^−6^ (10^2^ CFU/mL) were prepared in saline and 50 µL were inoculated onto BHI agar plates containing 4.0, 6.0, 8.0, 12.0 and 16.0 mg/L vancomycin and 4.0, 8.0, 16.0 and 32.0 mg/L teicoplanin. After 48 h of incubation at 37°C, colonies were counted and the log CFU/mL was plotted against the vancomycin concentration using GraphPad Prism software (GraphPad Software Inc., La Jolla, CA, USA). The ratio of AUC of the test isolates to the AUC of *S. aureus* Mu3 was calculated and was interpreted as described previously [43]. Mu3 (hVISA), Mu50 (VISA) and ATCC 29213 (VSSA) were used as control strains.

### Molecular characterization

Molecular characterization of MRSA strains included in the study was conducted by MultiLocus Sequence Typing (MLST), Staphylococcal Cassette Chromosome *mec* (SCC*mec*) typing and accessory gene regulator (*agr*) typing. All techniques were performed as previously published [44], [45].

### Susceptibility to Triton X-100 autolysis

Triton X-100 autolysis was assessed as described previously [46]. Briefly, mid-logarithmic phase cultures grown in BHI were assayed by rinsing bacterial cell pellets twice in ice-cold water followed by re-suspension in lysis buffer [0.05 M Tris–HCl pH 7.2, 0.05% Triton X-100 (Sigma)]. The decrease in absorbance (*A*_620_) was monitored at 30 min intervals for 5 h. The experiments were performed in triplicate and the results shown as the mean ± standard deviation of the autolysis ratio calculated as: O.D. T_0_/T_5_.

### Screening of δ-hemolysin activity on 5% sheep blood agar plates

The functionality of the *agr*-operon was measured by delta-hemolysin production testing the strain by cross-streaking perpendicularly to RN4220, which produces only β-haemolysin [47], on a sheep blood agar (SBA) plate. This test can usually identify the three staphylococcal haemolysins active on SBA – α, β, and δ–due to the interactions between them: β-haemolysin enhances lysis by δ-haemolysin, but inhibits lysis by α-haemolysin [49]. Delta-hemolysin produced by a test strain results in a zone of enhanced hemolysis in areas where this lysis overlaps with the beta-hemolysin zone of RN4420.

### RNA extraction, retro-transcription and quantitative real Time RT-PCR

An aliquot of an overnight culture was diluted 1∶50 and bacteria cells were grown in BHI at the exponential phase (OD_600_ = 0.4 at 3 h) in the presence of VAN and Ca^2+^-DAP, at the concentration equal to two dilutions below the initial MIC value, or in their absence, called “free condition” (F). Cells were then harvested by centrifugation, and the bacterial pellet was stored at −80°C until use. The cultures were re-suspended in 200 µl of diethylpyrocarbonate (DEPC) treated H_2_O, 1 ml of Trizol-reagent (GibcoBRL, Paisley, UK) was added and incubation continued for a further 5 min. Following incubation, 200 µl of chloroform was added, mixed by agitation; the mixture was incubated for 5 min, and centrifuged at 10,000 g for 15 min. After centrifugation, 1 ml of cold-isopropanol was added and the mix was maintained at −20°C for 60 min, followed by centrifugation at 12,000 g for 15 min and re-suspension of the pellet in 50 µl of DEPC-H_2_O and storage at −20°C. Genomic DNA was removed by treatment with Rnase-free-DnaseI (Ambion, Austin, TX, USA). RNA quality was determinated by analysis of the A_260/280_ ratio and analysis of the rRNA bands on agarose gels. RNA concentration was determined spectrophotometrically. Each extracted RNA sample was also checked for the presence of DNA contamination as a template in the PCR assay to confirm the absence of DNA contamination.

Retro-transcription, cDNA synthesis, was carried out by using the hexanucleotide primers ‘ImProm-II Reverse Transcriptase Kit’ (Promega) according to the manufacturer's instructions.

Quantitative real time RT-PCR was performed in a MX 3000P Instrument (Stratagene) with a 2.5 µl template (cDNA), the Brilliant SYBR Green QPCR Master mix (Stratagene), and 30 pmol of primers in a final volume of 25 µl.

PCR reaction efficiency was verified by using serial dilutions of cDNA ranging from 10^2^ to 10^6^ target copies per reaction (10^4^–10^8^ target copies per sample, standard curve), and only oligonucleotides giving PCR cycles which generate a linear fit with a slope between −3.1 and −3.6 and amplification efficiency value (Rsq) of 90–110% were chosen. All real time RT-PCR were performed in triplicate at an initial denaturation of 95°C for 10 min, followed by 35 cycles at 95°C for 1 min, 55°C for 1 min, 72°C for 1 min and a final cycle at 95°C for 1 min, 55°C for 30 sec and 95°C for 30 sec.

Primers for quantification were selected to amplify a fragment of less than 300 bp. *gyrB* was used as a normalizer, internal control, as previously published [50]. All the primers used for the real time RT-PCR study are listed in table S3.

The expression of the studied genes is represented as the increment/decrement (fold changes) of: i) hVISA and VISA isolates (Mu3-CZ1-SS33-Mu50-004/210) versus the VSSA isolate (NRS149), in drug-free conditions (F) (shown as a histogram or by fold-change values in supporting information tables); ii) each strain in culture with VAN or DAP with respect to the drug-free condition (shown as a histogram or by fold-change values in supporting information tables); iii) each strain versus all the others both in drug-free conditions and in media with the addition of VAN and DAP (shown as a relationship of the relative amount of transcripts of all strains in supporting information tables).

For each analysis, three to five distinct biological replicates were carried out.

Expression analyses were performed using the relative expression software tool REST2009 (Relative Expression Software Tool). REST applies the efficiency-corrected comparative CP method and performs randomization tests to estimate a sample's expression ratio and the likelihood of up or down-regulation, taking into account reference genes and the individual amplification efficiency of each gene. The Excel-based relative expression software tool, REST 2009, was applied for group wise comparison and statistical analysis of the qPCR data as described in Pfaffl *et al.* (http://rest.gene-quantification.info/). The relative expression ratios were calculated by a mathematical model, which included an efficiency correction for real-time PCR efficiency of the individual transcripts, as follows: Ratio = (E _target_) ^ΔCP^ target ^(control-sample)^/(E_ref_) ^ΔCP^ ref ^(control-sample)^.

The relative expression ratio of a target gene was computed based on its real time PCR efficiencies (*E*) and the crossing point difference (ΔCP) for an unknown sample versus a control. For each gene, cDNA dilution curves were generated and used to calculate the individual real time PCR efficiencies (*E* = 10^[−1/slope]^) [51], [52].

In particular, the purpose of this test was to determine whether there was a significant difference between samples and controls, while taking into account issues of reaction efficiency and reference gene normalization. The test uses the hypothesis test P(H_1_) that represents the probability of the alternate hypothesis that the difference between sample and control groups is due only to chance. The hypothesis test performs at least 2000 random reallocations of samples and controls between the groups. Statistical differences are significant when p<0.05.

### Sequencing and sequence analysis

All amplification products were purified using the QIAquick PCR gel extraction Kit (Qiagen) and sequenced with a LICOR DNA 4000L sequencer. The DNA sequence was analyzed by the gapped blast software [53].

## Supporting Information

Table S1 Relative quantitative expression of some autolytic, cell-wall charge and regulator genes in drug-free conditions.* The relative amount of transcripts was obtained statistically evaluating gene expression levels of each strain versus all the others.
(DOC)

Table S2 **Relative quantitative expression of some autolytic, cell-wall charge and virulence regulator genes with VAN (V) and Ca^2+^-DAP (D) versus drug-free conditions (F).** * The relative amount of transcripts was obtained statistically evaluating gene expression levels of each strain versus all the others.
(DOC)

Table S3 **Primer sequences of the studied genes.**
(DOC)
