# Supplementary material for: Modulating Activity of Vancomycin and Daptomycin on the Expression of Autolysis Cell-Wall Turnover and Membrane Charge Genes in hVISA and VISA Strains
Source: PLoS One. 2012 Jan 9;7(1):e29573. doi: 10.1371/journal.pone.0029573 (PMC3253798; doi:10.1371/journal.pone.0029573)
Supplement: Table S1 — Relative quantitative expression of some autolytic, cell-wall charge and regulator genes in drug-free conditions.* The relative amount of transcripts was obtained statistically evaluating gene expression levels of each strain versus all the others. (DOC) [file pone.0029573.s001.doc]

**Table S1.** Relative quantitative expression of some autolytic, cell-wall charge and regulator genes in drug-free conditions.

| **Gene** | **Product** | **Ratio of transcripts between strains of indicated combination as Mean of Fold Changes**  **(standard error)**  **(in bold significant values p<0.05)** | | | | | |
| --- | --- | --- | --- | --- | --- | --- | --- |
| **Autolytic genes** | | **CZ1**  ***vs***  **NRS149** | **SS33**  ***vs***  **NRS149** | **Mu3**  ***vs***  **NRS149** | **004/210**  ***vs***  **NRS149** | **Mu50**  ***vs***  **NRS149** | **Relative amt of transcripts**  **(p<0.05)** * |
| *atl* | N-acetyl muramoyl-L-alanine amidase | 0.73  (0.62-0.98) | 0.67  (0.57-0.93) | 0.70  (0.60-0.96) | 1.53  (1.19-2.06) | **0.59**  (0.48-0.79) | **NRS149=Mu3=SS33=CZ1=004/210>Mu50** |
| *lyt*M | Peptidoglycan hydrolase | 1.09  (0.97-1.26) | 1.04  (0.91-1.21) | 1.07  (0.95-1.24) | **0.15**  (0.09-0.28) | **0.36**  (0.27-0.51) | **NRS149=Mu3=SS33=CZ1>004/210=Mu50** |
| *sce*D | Trans-glycosylase | **1.85**  (1.73-2) | **1.82**  (1.70-1.97) | **24.25**  (20-29.6) | **5.49**  (3.51-9.51) | **19.33**  (15.2-23.5) | **Mu50=Mu3>004/210>SS33=CZ1>NRS149** |
|  | | | | | | | |
| **Cell wall charge genes** | |  |  |  |  |  |  |
| *mpr*F | Phosphatidyl- glycerol lysyltransferase | **3.28**  (3.08-3.65) | **3.25**  (3.05-3.62) | **5.96**  (4.87-7.34) | **5.92**  (4.69-7.48) | 2.08  (1.65-2.59) | **004/210=Mu3>SS33=CZ1>NRS149=Mu50** |
| *dlt*A | D-alanine-D-alanyl ligase | **0.34**  (0.26-0.42) | **0.31**  (0.230.39) | **0.30**  (0.22-0.39) | **0.62**  (0.46-0.75) | **2.23**  (1.66-2.98) | **Mu50>NRS149>004/210>Mu3=SS33=CZ1** |
|  | | | | | | | |
| **Regulator genes** | |  |  |  |  |  |  |
| *rna*III | agr-locus effector | **0.045**  (0.03-0.05) | **0.042**  (0.03-0.05) | **0.041**  (0.03-0.04) | **0.05**  (0.03-0.07) | **0.026**  (0.02-0.03) | **NRS149>Mu3=SS33=CZ1=004/210³Mu50** |
| *wal*KR | Two component regulatory system | **2.00**  (1.97-2.10) | **2.17**  (2.00-2.30) | **2.28**  (2.17-2.46) | 1.12  (0.8-1.2) | 0.76  (0.46-1.10) | **Mu3=SS33=CZ1>NRS149=004/210=Mu50** |
|  | | | | | | | |

* The relative amount of transcripts was obtained statistically evaluating gene expression levels of each strain versus all the others.
