# Supplementary material for: Modulating Activity of Vancomycin and Daptomycin on the Expression of Autolysis Cell-Wall Turnover and Membrane Charge Genes in hVISA and VISA Strains
Source: PLoS One. 2012 Jan 9;7(1):e29573. doi: 10.1371/journal.pone.0029573 (PMC3253798; doi:10.1371/journal.pone.0029573)
Supplement: Table S2 — Relative quantitative expression of some autolytic, cell-wall charge and virulence regulator genes with VAN (V) and Ca2+-DAP (D) versus drug-free conditions (F). * The relative amount of transcripts was obtained statistically evaluating gene expression levels of each strain versus all the others. (DOC) [file pone.0029573.s002.doc]

**Table S2.** Relative quantitative expression of some autolytic, cell-wall charge and virulence regulator genes with VAN (V) and Ca^2+^-DAP (D) versus drug-free conditions (F).

| **Gene** | **Product** | **Ratio of transcripts between strains of indicated combination as Mean of Fold Changes**  **(standard error)**  **(in bold significant values p<0.05)** | | | | | | | | | | | |
| --- | --- | --- | --- | --- | --- | --- | --- | --- | --- | --- | --- | --- | --- |
| **Autolytic genes** | | **Mu50D**  ***vs***  **Mu50F** | **Mu50V**  ***vs***  **Mu50F** | **Mu50D**  ***vs***  **Mu50V** | **Mu3D**  ***vs***  **Mu3F** | **Mu3V**  ***vs***  **Mu3F** | **Mu3D**  ***vs***  **Mu3V** | **Mu3D**  ***vs***  **Mu50D** | **Mu50V**  ***vs***  **Mu3V** | **NRS149D**  ***vs***  **NRS149F** | **NRS149V**  ***vs***  **NRS149F** | **Relative amt of transcripts**  **(p<0.05)** * | |
| *atl* | N-acetyl muramoyl-L-alanine amidase | **0.3**  (0.24-0.34) | **0.32**  (0.26-0.36) | 0.92  (0.80-1.10) | **0.053**  (0.04-0.06) | **0.053**  (0.04-0.06) | 1.01  (0.94-1.08) | **7.27**  (6.55-8.09) | **0.20**  (0.16-0.26) | **0.57**  (0.43-0.69) | **1.78**  (1.42-2.34) | **Mu3F>Mu3D=Mu3V**  **Mu50F>Mu50D=Mu50V** | **Mu3D>Mu50D**  **Mu3V>Mu50V** |
| *lyt*M | Peptidoglycan hydrolase | **0.27**  (0.19-0.47) | 0.76  (0.57-1) | **0.35**  (0.33-0.37) | **0.03**  (0.02-0.04) | **0.02**  (0.01-0.03) | 1.09  (1.01-1.18) | 0.36  (0.32-0.38) | 0.89  (0.68-1.15) | 1.05  (0.90-1.20) | **0.31**  (0.19-0.61) | **Mu3F>Mu3D=Mu3V**  **Mu50V=Mu50F>Mu50D** | **Mu50D=Mu3D**  **Mu3V=Mu50V** |
| *sce*D | Trans-glycosylase | **0.64**  (0.57-0.70) | **0.35**  (0.32-0.38) | **1.8**  (1.00-2.00) | **0.22**  (0.18-0.54) | **0.08**  (0.07-0.10) | **2.67**  (1.05-6.88) | **2.9**  (2.33-3.00) | **13.5**  (10.2-17.1) | **0.238**  (0.18-0.30) | 0.75  (0.55-0.93) | **Mu3F>Mu3D>Mu3V**  **Mu50F> Mu50D>Mu50V** | **Mu50D>Mu3D**  **Mu50V>Mu3V** |
|  | | | | | | | | | | | | | |
| **Cell-wall charge gene** | |  |  |  |  |  |  |  |  |  |  |  | |
| *mpr*f | Phosphatidyl-glycerol lysyltransferase | **11.03**  (9.96-12.45) | **0.31**  (0.26-0.35) | **34.37**  (29.81-40.73) | **1.61**  (1.47-1.76) | **0.34**  (0.32-0.36) | **4.79**  (4.37-5.20) | 1.16  (1.04-1.32) | 1.03  (0.88-1.28) | 0.33  (0.29-0.38) | 0.40  (0.34-0.47) | **Mu3D>Mu3F>Mu3V**  **Mu50D>Mu50F>Mu50V** | **Mu50D=Mu3D**  **Mu50V=Mu3V** |
| *dlt*A | D-alanine-D-alanyl ligase | **0.09**  (0.07-0.12) | **0.058**  (0.054-0.06) | **1.88**  (1.4-2.3) | **0.37**  (0.30-0.43) | 0.88  (0.73-1.04) | **0.42**  (0.37-0.48) | **0.22**  (0.17-0.3) | 0.98  (0.83-1.15) | **0.18**  (0.16-0.20) | **0.37**  (0.33-0.42) | **Mu3V=Mu3F>Mu3D**  **Mu50F>Mu50D>Mu50V** | **Mu50D>Mu3D**  **Mu50V=Mu3V** |
|  | | | | | | | | | | | | | |
| **Regulatory systems** | |  |  |  |  |  |  |  |  |  |  |  | |
| *walK* | Two component regulatory system | **0.26**  (0.15-0.51) | **0.37**  (0.27-0.60) | 0.70  (0.65-0.72) | 1.37  (1.28-1.44) | 0.56  (0.48-0.62) | 2.44  (2.2-2.6) | **15.81**  (10.81-29.00) | 0.66  (0.62-0.68) | 1.01  (0.88-1.24) | 1.21  (1.16-1.26) | **Mu3V=Mu3F=Mu3D**  **Mu50F>Mu50D=Mu50V** | **Mu3D>Mu50D**  **Mu50V=Mu3V** |
|  | | | | | | | | | | | | | |

* The relative amount of transcripts was obtained statistically evaluating gene expression levels of each strain versus all the others.
