# Supplementary material for: Modulating Activity of Vancomycin and Daptomycin on the Expression of Autolysis Cell-Wall Turnover and Membrane Charge Genes in hVISA and VISA Strains
Source: PLoS One. 2012 Jan 9;7(1):e29573. doi: 10.1371/journal.pone.0029573 (PMC3253798; doi:10.1371/journal.pone.0029573)
Supplement: Table S3 — Primer sequences of the studied genes. (DOC) [file pone.0029573.s003.doc]

**Table S3. Primer sequences of the studied genes.**

| **Primer** | **Gene** | **Sequence** | **Amplicon (bp)** | **Reference** |
| --- | --- | --- | --- | --- |
| CV102 | ***gyr*B** | 5’ CAACTATGAAACATTACAGCAGCGT 3’ | 256 | This study |
| CV103 |  | 5’ TGTGGCATATCCTGAGTTATATTGAAT3’ |  |  |
| CV110 | ***atl*** | 5’ GCTGTATCAGAATTTGGTGTTACATAG 3’ | 279 | This study |
| CV66 |  | 5’ CGGCTTATCAATGGTTCCTTG 3’ |  |  |
| CV122 | ***lyt*M** | 5’ CTATACATTCGTAGATGCTCAAGGAC 3’ | 228 | This study |
| CV123 |  | 5’ CGCTTGGTTGTTGTTACTATGTG 3’ |  |  |
| CV120 | ***sce*D** | 5’ CACCTGATGTTGGATTTACAGCA 3’ | 202 | This study |
| CV121 |  | 5’ CAATCACAAGAAGTTGAAGCACCA 3’ |  |  |
| CV130 | ***mpr*F** | 5’ GAACCACCGTTTTCAACTGAA 3’ | 244 | This study |
| CV131 |  | 5’GTAAATCTAACTCTGGCAACCATC 3’ |  |  |
| CV79b | ***dlt*A** | 5’ ATGTTTAGCATCAGGCGGTAC 3’ | 247 | This study |
| CV80 |  | 5’ACTTGGGAAACGGCTCACTAA 3’ |  |  |
| CV25 | ***rna*III** | 5’CTGAGTCCAAGGAAACTAACTCTAC 3’ | 235 | Cafiso et al.2007 |
| CV55 |  | 5’ TGATTTCAATGGCACAAGAT 3’ |  |  |
| CV114 | ***gra*R** | 5’GTTGCTGGTATTGAAGATTTCG3’ | 202 | This study |
| CV115 |  | 5’CGCCAAGTTCCATACTCATCAC3’ |  |  |
| CV116 | ***gra*S** | 5’CACCTGTGACAGCCATGAAATTA3’ | 186 | This study |
| CV117 |  | 5’CATCAATGACCATGCGTTTAAGTGACA3’ |  |  |
| CV142 | ***wal*KR** | 5’AAACAACTACAATCCCTTCATACTAA3’ | 250 | This study |
| CV147 |  | 5’CTTGACGGTTGGCATACTCACTTAA3’ |  |  |
